# The Gynæcological Society of Chicago

**Published:** 1888-07

**Authors:** 


### TRANSACTIONS OF THE GYNÆCOLOGICAL SOCIETY OF CHICAGO.

Regular Meeting, Friday, April 20, 1888.

The President, Henry T. Byford, M.D., in the chair.

The President exhibited a Uterus removed per Vaginam for Fibroids.

He said: I have here a uterus about double the normal size that I removed through the vagina on account of fibroid tumors. They had produced incurable stenosis, and were accompanied by almost constant pain of increasing severity. The patient, Florence Jones, is 42 years old, and has had four children, the youngest 20 years old. She is obliged to wash for a living, has been getting worse for two years, and during the past year has had to stop work every two or three days on account of suffering referred to the pelvis. She has been under treatment by prominent gynæcologists, both for the stenosis and for the fibroid tumors, without avail.

As nothing short of a severe cutting operation would have relieved the stenosis, I considered that vaginal hysterectomy would be but little less dangerous, and would give permanent and complete relief instead of temporary and partial. It would remove the tumors with but little danger and thus forestall a possible abdominal hysterectomy or supra-vaginal amputation with the terrors so often connected with them. You will notice that the whole uterus is enlarged and hardened, and that the cervix would form a bad stump in case of such amputation. Two of the tumors are near the serous surface, and one just under the mucous membrane. The stenosis extends half an inch above and a little below the internal os and is of cicatricial hardness. The tumors are each about the size of a hickory-nut.

As would be expected, the patient has not had a bad symptom, and will in two or three weeks be able to resume her place at the washtub.

The President said, in reply to questions, that the operation was performed five weeks ago, and the patient was ready to leave the hospital. The ovaries were not removed, as they were not diseased and were undergoing senile atrophy.

Professor James H. Etheridge reported the following Case of Cæsarean Section.

He said: I have not had time to commit to paper a description of this case, but I can give the salient points.

On the 21st of February, I was summoned to see a patient; I was notified that I was wanted to perform Cæsarean section; that there was a fluctuating tumor in the pelvis. I saw the patient about half-past ten in the morning, and upon examination I found the whole pelvis blocked up by a fluctuating tumor containing a hard substance. I could feel nothing else. The index finger could pass up between the symphysis pubis and the tumor, which seemed to be in the posterior part of the pelvis. I could pass one finger easily, but I could not get two in, and with the utmost pressure of my hand upwards I could not reach the cervix.

The patient was an Irishwoman, 32 years of age, of nervous temperament, and had borne three children. The first labor was normal. The second labor was terminated by instrumental delivery with some difficulty, and the third one, two and a half years ago, was accomplished with the greatest difficulty, with forceps. There was an obstruction to delivery found at that time, but what it was was not determined. The next thing known of her was she fell in labor, and the attending physician upon examination detected what he communicated to me. I could not determine what the fluctuating tumor was, and the idea of puncturing it occurred to me upon my first examination, but fearing that I would open a pus cavity that would discharge its contents during the lying-in period, I was deterred from doing it. After a hurried consultation it was decided that the patient should be taken to the Presbyterian Hospital. She reached the hospital at a quarter-past one. The attending physician went with her in a carriage and administered chloroform *in transitu*. About half-past four she was etherized and the initial incision made from above the umbilicus, perhaps an inch and a half, down to the probable location of the reflexion of the peritoneum upon the uterus. The abdomen was very large, and in making the first incision the edge of the scalpel went through the abdomninal wall and into the uterus itself. I supposed I was making a cutaneous incision, but the wall was so thin that the knife went through into the uterus. The steps of the operation after that were very simple. The incision was enlarged to about six inches, perhaps seven. The amniotic sac was not broken, and two or three sweeps of the knife carried it through the uterine wall and into the bag of waters, and there was a tremendous welling up, the water flowing out over the patient, the table, and everything else. Dr. Parkes was my vis-à-vis, and he immediately pressed the uterus laterally with the view of forcing the child up through the opening, and it came up breach first. There was no attempt of the uterus to contract. The child was easily taken out in a second of time, and two snap forceps put upon the cord, which was divided and the child given to the nurse. There was very copious hæmorrhage, though not alarming; the walls on both sides seemed to be springs of blood. The contraction of the uterus was secured by the pressure over each side, no attempt being made to turn the uterus out of the cavity. The placenta was easily secured; the peeling off of the whole of it from the inside of the uterus was speedily and easily and cleanly accomplished. Uterine contractions failed to follow immediately. A hypodermic injection of ergot was given and snap forceps put on bleeding vessels until the hæmorrhage was controlled. The uterus was manipulated and pinched and every effort made to invite nature to set up contraction, which, after a space of seven or eight minutes, came on feebly, not vigorously.

After the uterus was thoroughly emptied, I passed my hand down into the cervix to ascertain if it was patulous, and I found all four of my fingers could go through easily up to the second joint, showing that there would be an outlet for the lochial flow. The appearance of blood at the vulva indicated that drainage from the uterus would be all that could be desired.

The closure of the uterus was accomplished by three rows of sutures; the first one, which brought together the mucous membrane, was made with a carbolized sllk ligature. A larger ligature was used for the muscularis. A fine silk suture was used for the peritoneal covering of the uterus. In the mean time the uterus was contracting very steadily indeed, and the hæmorrhage was well under control, so that by the time the muscular wall was brought together it was a dry wound. The abdominal wall was closed in the ordinary way with interrupted sutures and the patient was put to bed with stimulants and hot applications, and she reacted very well. She did well for twenty-four hours without any particular rise of temperature, but in the second twenty-four hours there was a gradual coming up of the temperature and peritonitis set in. I wished to take advantage of cathartics and get her bowels open, but every effort that was made to physic her, in the second twenty-four hours, was entirely futile. Injections by the rectum were attempted, but the pressure upon the rectum prevented the introduction beyond the sphincter ani of a rectal tube; in the mean time the temperature was going up and the patient getting weaker. Then it was decided to open the fluctuating tumor in the pelvis and see what it was. The patient was chloroformed and placed in the extreme lithotomy position. The posterior vaginal wall was found to be extremely blue. The

Cæsarean section was performed about four o'clock in the afternoon, and this operation was done near the close of the third twenty-four hours afterwards, viz., about two o'clock. An incision was made in this bulging mass and the pus welled out in great quantities, amounting to about half a gallon. Upon introducing the index finger into the abscess cavity, a dermoid cyst was detected, the hair being easily detectable. The patient was put to bed after thorough evacuation of the pus, but she never rallied from the shock of the operation and died in twelve hours.

It occurred to me, on first examining her, that this could not be an accumulation of ascitic fluid, and that it must be pus. From the history of the case and the deduction that was speedily made, the decision was reached that abdominal section should be made and the child removed in that way, because in the examination one could feel up in the vagina, with the finger, a hard mass of something below the promontory of the sacrum that appeared to me would render it impossible to introduce forceps and to drag the child through ; and if it was filled with pus, I was certain the woman would die of infection.

Professor W. W. Jaggard read the following paper, entitled "A CASE OF CONSERVATIVE CÆSAREAN SECTION UNDER THE RELATIVE INDICATION, WITH TERMINATION IN RECOVERY."

I desire to place on record the following case of conservative Cæsarean section performed under the relative indication, with termination in recovery. In passing, I beg to call attention to certain points of practical interest in connection with the measurement of the pelvis, the indication for, and the technique of, the operation.

Dr. Patrick Dougherty, of Chicago, a short time since invited me to see in consultation a case of alleged contracted pelvis. We examined the patient in the third week of February last, and elicited the following history.

*Case.*—Mrs. E. S., 36 years old, born in Hillesheim, in the region of the Eyfel Gebirge, Rhenish Prussia, married in the United States shortly after immigration. She had been a sickly child, unable to walk until her seventh year, on account of *Doppelglieder*, *i e.*, rachitis. During infancy, she suffered from tuberculosis of the cervical glands, two depressed cicatrices being visible on the left side of the neck at the time of examination. Since her seventh year, she has enjoyed robust health.

Her mother gave birth to four children, three of whom were females. All of these labors were normal. Of the patient's two sisters, one has had normal confinements, while the other has been invariably delivered by the aid of instruments.

First pregnancy : patient's first child was delivered May 13, 1882. Shoulder presentation, right scapula anterior position. Difficult delivery by version, decapitation, and extraction. Puerperium normal.

Second pregnancy, delivery June 20, 1883. Same presentation and position as in first pregnancy. Prolapsus of funis. Delivery by version, extraction, and forceps to the after-coming head. Septicæmia, puerperium six weeks.

Third pregnancy, induction of premature labor at the end of the seventh lunar month. Same presentation and position as before. Delivery by version and extraction. Child survived the difficult operation a few hours. Puerperium normal.

Fifth pregnancy, beginning of last menstruation June 1, 1887. *Status præsens:* The patient, of strong frame and well developed muscles, is four feet seven inches in height, and one hundred and thirty-five pounds in weight. Pregnant; near term; distance from ensiform cartilage to pubis, forty-five centimetres ($17\frac{1}{2}$ inches); from ensiform cartilage to umbilicus, twenty-two centimetres ($8\frac{3}{4}$ inches); circumference around umbilicus, eighty-seven centimetres (34 inches). Shoulder presentation, right scapula anterior position.

*Pelvic Measurements.*

| | | |
|---|---|---|
| Distance between anterior-superior spinous processes | 27 cm. | ( 10½ in.) |
| Distance between iliac crests | 27 cm. | ( 10½ in.) |
| External conjugate diameter (Baudelocque) | 14 cm. | ( 5½ in.) |
| Distance from sacro-coccygeal joint to subpubic ligament (A. G. E. Breisky) | 9 cm. | ( 3½ in.) |
| Distance between the great trochanters | 30 cm. | (11.7 in.) |
| Pelvic circumference (Kiwisch) | 85 cm. | ( 33¼ in.) |
| Diagonal conjugate diameter | 7.5 cm. | ( 2.9 in.) |
| True conjugate diameter (estimated) | 5.5 cm | (2.14 in.) |

*Diagnosis.*—Simple, flat rachitic pelvis, with so-called absolute contraction of the true conjugate diameter. Apart from the pelvis, the osseous system showed no marked signs of rachitis. There was no abnormal spinal curvature, antero-posterior or lateral, and the long bones were perfectly straight.

*Indication for Operation.*—Notwithstanding the fact that the pelvis was a typical example of the so-called absolutely contracted simple, flat, rachitic class, the history of former deliveries demonstrated plainly that the obstacle to the escape of the child through the natural passages was only relative, and not at all insurmountable. Nor is it necessary, in order to explain the woman's survival of former labors, to invoke extraordinary skill upon the side of the medical attendants—in all twelve in number—nor unusual physical endurance upon the part of the patient, although both conditions were doubtless supplied. Both parents were undersized, with relatively small heads, and the children were of a size less than is common. Moreover, the after-coming head was invariably made to present, and the accommodation of the passenger to the passages was thus greatly facilitated. The case was clearly one in which the woman could be delivered with safety, in all probability, by version, extraction, and craniotomy. On the other hand, the child was living, and Cæsarean section offered the possibility of saving both mother and child, although, of course with enormously increased maternal risk. The question of the induction of premature labor, so late in pregnancy, was not considered for obvious reasons. In a word, the relative indication for Cæsarean section was presented.

The most important condition upon which this indication depends at the present time is the consent of the woman, obtained without direct or indirect coercion. Accordingly, a plain, unvarnished statement of all the facts in the case was made to the patient. She was clearly and distinctly informed that, by the destruction of the child and its removal as in former pregnancies, her life would be almost certainly saved, and that the attempt to save both lives by Cæsarean section would be attended by enormously increased danger to herself. After a week's deliberation, she elected the Cæsarean operation. In reaching this conclusion, she was assisted by the Roman Catholic priest of the parish. This gentleman remarked that the pregnant woman was the aggressor; that she had made the contract of maternity; the child was passive and had made no contract. In strict equity, entirely apart from ecclesiastical considerations, the child's claims to life should be considered at least equally with those of the mother.

*Operation.*—The patient at once entered Mercy Hospital. The urine was examined and found to be normal. The only preparatory treatment consisted in a daily bath, in tepid water, with the liberal use of soap, which the woman's mode of life before admission rendered necessary.

In the selection of the time for operation, I had determined to choose the latest possible moment before labor actually began. From the usual data—date of last menstruation, size and position of the uterus, abdominal measurements, length of the child measured by calipers (Ahlfeld), estimate of the size and weight of the child by palpation (Carl Braun)—it was possible in this case to make only a probable diagnosis of the time of gestation. I concluded that the woman was in the last fortnight of pregnancy.

Early Tuesday morning, March 6, the patient informed me that she would certainly fall in labor within the next twenty-four hours. She based her prediction upon dull pain referable to the lumbar and sacral regions, and beginning painful uterine contractions. She had been enabled to foretell her other confinements by similar sensations, and I was inclined to attach considerable importance in this case to subjective signs. The only objective symptom indicative of impending labor was a slight increase in the force and frequency of the intermittent uterine contractions. So the hour for the operation was fixed upon at one in the afternoon.

All precautions were taken with respect to the most thorough cleanliness and disinfection of the operator, assistants, patient, instruments, and environment.

Dr. W. E. Casselberry administered the anæsthetic (ether); Dr. E. C. Dudley, Dr. Bayard Holmes, Dr. G. W. Whitfield, Dr. B. L. Riese, Dr. M. Scheuer assisted me in the operation; Dr. Patrick Dougherty and Dr. Charles Caldwell assumed charge of the babe. I take this opportunity to make my grateful acknowledgments to these gentlemen for their efficient services.

The woman was in excellent condition; cheerful; pulse and temperature normal.

The steps in the operation were: After evacution of the bladder, incision through the linea alba, from the navel to a short distance above the pubes, as low down as was safe, on account of the bladder. The diastasis of the recti muscles was well marked, and the peritoneum was incised without dividing much muscular tissue. No omentum nor intestines presented between the uterus and anterior abdominal wall.

The median line of the uterus coincided with the incision, and the usual manipulation to correct lateral version and axial rotation was unnecessary. Before making the uterine incision, Dr. Holmes placed one hand on either side of the cut, and rendered the abdominal parietes tense enough to prevent the access of fluid to the peritoneal cavity. I incised the anterior uterine wall in the median line at a point a short distance above the os internum with a scalpel, and rapidly enlarged the cut in the direction of the fundus, to the extent of thirteen centimetres (5 inches) with a blunt-pointed bistoury. The thickness of the uterine wall was about one centimetre (one-third of an inch.)

The placenta was implanted over the line of incision, and the first gush of blood was frightful. The after-birth was quickly separated by the hand, the amnion ruptured, the child caught by the feet, turned, and delivered without laceration of the uterine wound. The child uttered a lusty cry upon its liberation from the cavum uteri. I had requested an assistant to insert his index fingers into the upper and lower angles of the uterine incision, and bring them up close to the abdominal cut as an additional precaution against the escape of fluid into the peritoneal cavity. In the hurry of the operation, this request was forgotten. After, or, rather, during the evacuation of the uterus, Dr. Holmes pressed this organ through the abdominal incision, by his hands applied on either side, while Dr. Riese brought the edges of the abdominal cut together behind the uterus, and effectually prevented all intestinal protrusion. The lower uterine segment, after this eventration, was firmly compressed by Dr. Holmes with the thumbs and index fingers of both hands, while the corpus uteri was enveloped in hot sterilized gauze compresses. Squibb's aqueous extract of ergot was exhibited hypodermically after the evacuation of the cavum uteri.

Hæmorrhage was trifling after the contraction and retraction of the uterine musculature, following the escape of the fœtus and envelopes, and was now fully controlled by digital compression. The elastic ligature was not used in the operation.

Twenty-one deep uterine sutures were inserted, including all the tissues down to the mucosa. For the introduction of these sutures, I used the long, slender laparotomy needle of Thomas Keith. This needle passes with remarkable ease through the thick uterine wall, making a very small puncture that is completely filled up with the suture material—in this case silk. After passing a finger through the canal of the cervix from above downward, the uterine cavity was irrigated with a five-per-cent. solution of carbolic acid, a bacillum containing ninety grains of iodoform placed within, and the wound closed. Union of the peritoneum over the line of incision was effected by a continuous silk suture. When the two rows of sutures had been drawn taut, the uterine wound was accurately closed, and perfectly dry. The uterus, in a state of normal retraction, was returned to the cavity of the abdomen.

The toilet of the peritoneum was brief, as no fluid had escaped into the abdominal cavity, and the intestines had not at any time protruded. The abdominal incision was closed with interrupted silk sutures.

The duration of the operation was about one and one-quarter hours. From the extraordinarily simple *technique*, it would seem that the operation had been needlessly prolonged. But the uterine sutures were inserted deliberately and with care; then, too, time was occupied in securing uterine retraction by the application of hot compresses.

The total amount of blood lost was not great—scarcely more than the average loss in normal labors. The chief element of danger lay in the suddenness of the loss, but no indication arose for the employment of transfusion, the apparatus for which was in readiness.

The shock from the operation was profound, but brief. The patient fully reacted within three hours. Her convalescence was uninterrupted. The pulse at seven, the day of the operation, was rapid, 120 beats to the minute, tense and small. It became gradually less frequent, less tense, until at the expiration of the first week it was normal. The temperature remained nearly normal, showing slight variations in the second week. These variations were attributed to several severe burns, suffered as the result of the injudicious application of hot bottles immediately after the operation. On the third day, the audible escape of flatus was noted, and about the same time the patient began to void urine spontaneously.

Tympanitis was notable by its absence throughout the recovery. On the fifth day the bowels were painlessly evacuated after the exhibition of citrate of magnesia, for which a preference was expressed.

The patient did not vomit at all, not even when she was recovering from anæsthesia.

The lochial discharge was slight, odorless, and ceased at the expiration of two weeks. Lactation was not established. After all former confinements the milk secretion was abundant.

Examination to-day, April 20, reveals the uterus nearly normal in size in mobile anteflexion. The parametrium is free from any sign of infiltration, and no trace of the sutures in the anterior wall of the uterus can be felt upon careful bimanual exploration. The vaginal finger easily outlines the anterior aspect of the uterus. The uterus is situated relatively high up in the pelvic cavity, but can be readily made to descend below the place of the inlet by gentle pressure above the pubes. I suspect the presence of adhesions—they must be very slight, however—between the fundus and the anterior abdominal wall.

The child was a small, but perfectly formed, and apparently mature male; weight, 3,000 grammes; length, 49 centimetres. The infant thrived on artificial feeding until the sixteenth day, when, after exposure to cold, it died suddenly in a convulsion. The autopsy disclosed intense pulmonary congestion. Although the child was apparently well nourished, it is not improbable that inanition was a predisposing factor. The death of the child was a matter of regret, apart from other considerations, on account of the possible unfavorable influence on the mother. However, she bore the loss calmly, feeling happy that she had given birth to a living child, capable of baptism.

The diameters of the fœtal head were:

Occipito-frontal . . . . . 11 cm. (4.29 inches).
Occipito-mental . . . . . 12 cm. (4.68 inches).
Bi-parietal . . . . . . . . . . 8¾ cm. (3.4 inches).

*Pelvimetry.*—Dr. R. P. Harris, whose eminent services as the statistician of Cæsarean section are universally recognized, writes in a recent communication to the *Medical News*, March 31, 1888: "What is wanted now is a better acquaintance with pelvimetry, and the steps of the improved operation, as it is performed in Leipzig, Dresden, and New York." No one doubts the truth of this proposition. Certain it is that the notion of pelvimetry generally entertained is obscure and confused in the extreme. Dr. E. C. Dudley informs me that a few weeks ago he encountered a case in which a wife, desirous of becoming a mother, confessed to the practice of the prevention of conception through a period of ten years, under the advice of two distinguished practitioners, upon the ground of alleged contracted pelvis. Careful measurements revealed the fact that the pelvis was unusually large. The woman has since become pregnant. But it is needless to multiply examples of such irresponsible opinion, when we have fatal ignorance flippantly displayed in the literature of the subject. A very pernicious book by a very excellent man, and published only two years ago, contains the following sentence: "External pelvimetry, while of undoubted service in large averages, is of no use in an individual case. The most common application of it is to measure the conjugate diameter by means of Baudelocque's calipers or the like instrument. One point of the calipers is placed on the back over the sacrum, the other over the symphysis pubis, and the distance between is noted. We then guess how thick the sacrum and dorsal tissue are, and how thick the symphysis must be, and, deducting these measurements, we can guess how long the conjugate diameter is, which might have been done without so much trouble in measuring." Many of the cases of Cæsarean section recorded in American annals are rendered well-nigh valueless for the purposes of comparative study by the omission of accurate pelvic measurements. Now, it must be admitted that the exact determination of the size and form of the pelvis constitutes one of the most difficult problems in obstetrics. A survey of the enormous mass of literature upon this subject accumulating since the discovery of the contracted pelvis by Julius Cæsar Arantius three hundred years ago, fully confirms this opinion. As an excellent critical, historical review of the subject, I beg to recommend to the Fellows of this Society the monograph* of Dr. Felix Skutsch. While all methods of pelvimetry fail to yield absolutely accurate measurements, and our notion of the pelvic anomaly in the concrete case must be inexact to a degree corresponding, still the diameters and dimensions just mentioned are amply sufficient to establish the probable diagnosis of the shape and relative size of the pelvis in the individual case of the more usual types of deformity, and to afford data for comparative study, and ground for action.

I append the corresponding normal diameters and dimensions, as given by Carl Braun and Schroeder:

* "Die Beckenmessung an der lebenden Frau." Jena, Gustav Fischer. 1887.

Distance between anterior superior spinous processes............26 cm.
Distance between iliac crests.....29 cm.
External conjugate diameter (Baudelocque)......... ..........20¼ cm.
Distance from sacro-coccygeal joint to subpubic joint (A. G. E. Breisky) .....................12.3 cm.
Distance between great trochanters 31½ cm.
Pelvic circumference (Kiwisch) ..90 cm.
Diagonal conjugate diameter.....13 cm.
True conjugate diameter ........11 cm.

II. *The Relative Indication for Cæsarean Section.*—Of course, as an operator, I was more pleased to perform Cæsarean section than to do craniotomy. But the humor of the medical attendant sustains no relation to the ethics of the case. I cannot forbear to reiterate here certain convictions that must always come up for consideration in similar cases. These propositions I beg to submit, if the expression be not too harsh, not so much as matters of opinion as matters of fact.

1. The necessary maternal mortality of craniotomy, performed under the conditions demanded in Cæsarean section as respects freedom from exhaustion and infection of the patient, with the best instrument and adequate skill, in cases of the simple, flat, rachitic pelvis, with a *conjugata vera* of six to eight centimetres, is zero. The simple, flat, rachitic pelvis is used as a type in this thesis on account of its relatively frequent occurrence. In the generally contracted and flat pelvis a *conjugata vera* greater than six centimetres must be postulated unless, as in the case I have just reported, the fœtal head is uncommonly small. It has been reserved for Leopold to demonstrate the truth of this proposition. While in 215* cases of craniotomy collected from the records of the Berlin Polyclinic, the Clinic at Halle, and the Leipsic Polyclinic, the entire maternal death-rate was 5.6 per cent., the total maternal mortality in Leopold's Clinic at Dresden during the interval, 1883–1887, after craniotomy, including 71 cases,* was 2.8 per cent. In these two fatal cases the cause of death was eclampsia, so that the mortality due to the operation itself has been reduced to zero.

The operation, performed under its own peculiar conditions, with the best instruments, is not extraordinarily difficult. It does not imply a higher degree of operative skill than it is fair to presume every qualified practitioner possesses. I have observed in all about thirty cases of craniotomy, and have never noted especial difficulty in the technique of the operation, nor unfavorable results to the mother, when the procedure was really indicated, and when the necessary conditions were present.

On the other hand, the mortality of conservative Cæsarean section, even when the necessary conditions have been supplied, is still considerable. Of Leopold's twenty-three cases of the improved Cæsarean section, two, or 8.4 per cent., died. The following extract from a letter recently received from Dr. Robert P. Harris is of interest in connection with American statistics:

"Your case makes sixteen Sänger-Cæsarean sections for the United States, with seven recoveries; and 165 for the whole Cæsarean list, with sixty-three women saved.

"I have twelve cases on record for the the last fifteen months, with six women and nine children saved; yours makes the thirteenth. There were eight operations in 1887, all Sänger's but one, with four women and five children saved. I have three cases in already for this year: one each for January, February, and March; one woman and three children saved."

The Cæsarean section is, and must always remain, the most difficult, dangerous, and formidable procedure in operative obstetrics. The shock incident to the opera-

* Wyder, Archiv f. Gyn., Bd. xxxii., Hft. 1, p. 60.

* Leopold, "Der Kaiserschnitt," etc., Stuttgart. 1888.

tion, entirely apart from sepsis and the loss of blood, is an element of danger that can never be completely eliminated. It is, perhaps, needless to remark that the successful performance of this operation does imply such a high degree of operative skill, and such an experience in this particular operation, as it is fair to presume the average practitioner does not possess.

As remarked by Leopold,* "The time has not yet arrived when craniotomy upon the living child can be unconditionally substituted by Cæsarean section. In a good many cases perforation may be avoided, and in a still larger proportion it cannot be dispensed with."

And Praeger † draws this important conclusion, "In cases presenting the relative indication, and which in a hospital might be subjected to Cæsarean section, the general practitioner, as a rule, ought only to consider craniotomy as the operation involving least risk to the mother."

2. The consent of the patient, obtained without direct or indirect coercion, is an essential condition to the relative indication. The time will probably come when under certain circumstances—*ex. gr.* in hospital practice—the woman shall not be permitted to elect as freely as she must be allowed to do at present.

3. The life of the adult female, who has already contracted relations with society, is of incomparably greater value, as judged by human standards, than the problematical existence of an unborn babe. Moreover, the expectancy of life in such children is decidedly less than in children of normal birth. If the operation is performed before the objective changes of labor are evident, as in the case under discussion, there is the risk of the premature interruption of pregnancy, of obviously serious prognostic moment with reference to the child. The necessary early ligature of the cord deprives the infant of an average amount of blood of ninety-two grammes (Budin, Ribemont). The mother is seldom able, even if she were to be permitted, to suckle her child. Finally, the offspring of women, affected with rachitis or osteomalacia, are frequently feeble, sickly, and unable to resist the unfavorable influence of the environment, entirely apart from the effect of hereditary disease. It is not my intention to use the death of the child in this particular case as an illustration of the truth of the statement just made, since in my judgment that event occurred chiefly as the result of most gross carelessness, *i. e.*, exposure of the child before an open window on one of those bitterly cold days in the latter part of March. Of the twenty-three children delivered alive by Leopold, one died a few hours after the operation (neglected shoulder presentation, laceration of the liver), eight died principally from cholera infantum within from three weeks to one year of the operation, eleven were living at the expiration of one year. The fate of three is unknown.

Now, while the present status of the Cæsarean operation with us scarcely justifies the words of Mauriceau, aptly quoted by Professor Lusk,* "If it be true that any women have escaped, it was the work of a miracle, or the express wish of God, who, if he wills it, is able to raise the dead, as he did Lazarus"; still it does suggest the often quoted remark of Cazeaux: "That which is certain respecting the Cæsarean operation is that more than the half of the women are immediately sacrificed, and that which has been well proven by the experience of the centuries is that, supposing all the infants alive at the moment of their birth, we will see not more than one-half attain the age at which their mothers succumbed."

I do not wish to be regarded as an obstructionist, but desire merely to utter a voice of warning. In this "Cæsarean Revolution in Progress in the United States," let us go

* "The Prognosis of Cæsarean Operation." The Medical News, October 8, 1888, p. 412.

slowly. In the words of Professor Cameron, of Montreal, that I quote from a letter, and without his permission, "Too much has been claimed for the section; a reaction is bound to set in ere long." Draw the lines of indication and condition more exactly, and surrender the operation to a special class of practitioners.

III. *The Operation.*—The items of special interest in connection with this particular case of Cæsarean section, are:

1. In the selection of the time of operation, I acted upon Schroeder's advice, and chose the latest possible moment before labor actually began. The advantages of an aseptic genital canal, daylight, adequate assistance, and the like, outweigh the danger of atony after evacuation of the uterus. The researches of J. Braxton Hicks * on "The intermittent contractions of the uterus during the whole of pregnancy" are perfectly familiar to the English-reading profession. In a recent note, this observer writes, "These intermittent contractions, always going on, are ready to be intensified by any exciting cause, and especially so at the periods of the suspended menstruation." . . . "The rapidity with which labor can be induced at almost any time of pregnancy is explained now quite readily. Formerly the fact was not explained; indeed, the time for termination of delivery can be precisely stated—the whole process done to order, as Dr. Robert Barnes and myself have pointed out. Dilate the os by elastic bags, turn the fœtus by my method, and in two hours generally the fœtus is expelled. The whole need not occupy more than than from six to eight hours." I was present some years ago at a Cæsarean section performed shortly before term by Professor Spaeth, assisted by Dr. Lumbe and Dr. Ehrendorfer. In this case, as in my own, there was no difficulty in securing retraction after the evacuation of the viscus. Of course, one runs the risk of moment to the child—of interrupting pregnancy some time before term, since only an approximate estimate of the time of gestation can be made from the data we can at present command.

*The Lancet, p. 554, March 17, 1888.

2. The uterus was incised *in situ* and the liquor amnii evacuated through the abdominal cut. Leopold recommends the eventration of the uterus before incision, and it is the common custom to rupture the amnion *per vaginam*. Sänger in his paper, read at the International Medical Congress, recommends the course pursued in this case. I had confidence in the ability of Dr. Holmes' hands to keep blood and liquor amnii out of the peritoneal cavity, and a short cut is of obvious advantage in retaining the intestinal mass within the abdominal cavity, not to mention other benefits.

3. Hæmorrhage was controlled by the normal tonus of the uterus and digital compression of the lower uterine segment. I did not apply the elastic ligature around the lower uterine segment before or after the uterine incision, on account of the danger of paralysis of the structures at and below the point of compression, a danger to which Sänger,* Doléris, and others have called attention. The amount of blood lost during the incision need not be much greater *without* than *with* the elastic ligature. It need not be much more than the amount of blood in the uterus at the time the incision is begun, provided subsequent procedures are executed quickly. The quantity of blood in the uterus at the time the incision is begun is necessarily lost.

4. The long laparotomy needle of Thomas Keith rendered the closure of the uterine wound easy and comparatively rapid. The puncture is very small, and is completely filled out by the suture material.

5. The suture material used in this case was silk. The influence of the suture material on the functions of the uterus, menstruation and pregnancy, is a question of grave practical moment. Leopold has

*Transactions of Ninth International Medical Congress.

rejected silver wire entirely and prefers chrome catgut to silk. In nine consecutive cases he has used this material with entire satisfaction.

The superficial uterine suture was intended to effect linear union of the incised peritoneum, and no attempt was made to fold that membrane into the divided muscularis in order to oppose peritoneal surfaces of relatively great areas. This constitutes a departure from Sänger's method. Schroeder* pointed out the essential weakness in the sero-serous suture, when he called attention to the fact that incised wounds with their edges accurately approximated are surer to heal than opposed peritoneal surfaces. In order that opposed peritoneal surfaces should unite, some new irritation is necessary to produce adhesive inflammation. This observation has since been confirmed by the investigation of Zweifel, Graser† and J. Veit.‡

Professor C. T. Parkes: As I was present at Dr. Etheridge's operation, I should like to say a few words in regard to some of the impressions made upon me by the operation. First, in regard to its severity; so far as my experience goes with disease or injury affecting the abdominal cavity, I am sure I have been through cases quite often that would give me a great deal more anxiety and be found more troublesome to manage than the doing of this operation. It struck me as being a very simple operation, at least to any one who is accustomed to having anything at all to do with the abdominal cavity. In the first place, the uterus is so large and it fills up so much of the cavity of the abdomen, that all the other viscera are out of the way, as was beautifully illustrated by Dr. Jaggard; there was nothing to be seen when the abdominal cavity was opened except the uterus, and it was very easy for the surgeon to open it. It was one of the easiest things imaginable to get the contents of the uterus out. So far as the flow of blood was concerned, I did not think there was any great amount of blood lost, considering the tissues divided and the size of the vessels cut. The gush is at first rather astonishing, but after a little time there is a rapid cessation of the bleeding. The bleeding in this case was very readily controlled by compression, and it did not strike me as being hurtful to the patient. I am quite sure she did not show any of the signs that are so often present when a large amount of blood is lost in other parts of the body. I am sorry that a complete post-mortem was not made after the death of the patient because I was very desirous of seeing the result of the manner of suturing that was adopted, because it struck me as being perfectly safe. After the suturing was done, the wound was perfectly dry in every way, and after this part of the work was over and the operation done, with the exception of closing the abdominal wound, no sponging was required. There seemed to be no trouble in keeping everything out of the abdominal cavity, either amniotic fluid or blood.

Another point struck me in this case, not directly in connection with Cæsarean section, but as interesting in obstetrics. I have always been taught and always supposed that, during the development of the uterus in the latter stages of pregnancy, all parts were dilated, that the cervix was opened out and became part of the body, that it was thinned out so that the cervix and body became continuous, but to my astonishment, when the body of this uterus was exposed, the cervix was perfect in shape and retained the same relation to the body as in the normal state. There did not seem to be any losing of the cervix in the body by dilatation.

The President: I congratulate Dr. Etheridge upon his courage in performing Cæsarean section under such adverse circumstances. Most operators would have preferred to attack the pus tumor from

---

*Zeitschr. d. Geb. und Gyn., Bd. 10, 395.

†Habilitationschr., Erlanger, 1886.

‡Deutsche Med. Wochenschr., 1888, No. 17.

below, delivered the child through the vagina, and have trusted to antiseptic treatment to prevent or modify subsequent septicemia.

I wish also to congratulate Dr. Jaggard upon the completeness of his paper in placing this subject before us in its most modern aspect.

Dr. J. C. HOAG: I do not feel like saying anything after so many others have maintained silence, but I think the papers deserve to be favorably discussed, and at least some questions might be asked on the subject. I thought, while the papers were being read, of a medical meeting I attended some time ago in London, where the relative merits of Cæsarean section and craniotomy were discussed. The meeting was attended by some of the most eminent men of England, and there seemed to be a very general consensus of opinion that craniotomy should be entirely abandoned. It seemed that the discussion first arose at a meeting of the British Medical Association, and it was afterwards kept up for some time in the society to which I refer. I was a little astonished to find that, at the same time the opinion was prevalent that craniotomy (embryotomy was perhaps the term used) should be abandoned, there was not a gentleman present who knew what Sänger's operation was, and it was suggested then that some one look up the subject and report at the next meeting.

With regard to the technique of the operation I remember to have read that on one occasion, when there was quite profuse hæmorrhage and where retraction and contraction did not take place after the operation, the operator was bold enough to keep the uterus open for an hour and a half, keeping it packed with ice, and that the patient made a good recovery. I have also noticed somewhere that some operator has suggested the advisability of putting in a few sutures in the abdominal wound previous to incising the uterus, so that after removing the child the walls might be drawn together somewhat and thus aid in the prevention of accumulations in the peritoneum. I would like to hear what the gentlemen think of these suggestions.

Professor F. C. SHAEFFER: I think the essayist deserves great praise for his courage, and is to be congratulated upon the brilliant results. Considering the varied circumstances under which these operations were done, it seems to me the results were extraordinarily good. It has afforded me very great pleasure to hear these papers to-night.

Professor CHARLES WARRINGTON EARLE: I would like to ask Dr. Etheridge one question: Were you able, in the time at your command, to make any effort at trying to find, through the incision in the abdominal wall, the nature of that obstruction below? You knew there was a fluctuating tumor there; did you examine it at the time of the operation?

Professor ETHERIDGE: I did not. I was so anxious to close up everything and get it out of the way that I did nothing of the sort. I was very greatly disappointed in our inability to secure a post-mortem examination. The next morning, when I reached the hospital, about half-past nine, the body had been taken away, so that that part of it will always have to remain obscure.

In regard to doing the other operation of opening the abscess and letting the discharge come away from it, I can say this: I have my doubts as to the possibility of saving the woman under the circumstances, from the fact that the pelvic cavity was not sufficiently enlarged by the evacuation of the tumor to admit of pulling the child through. This still constitutes, in my mind, an imperative objection to opening the tumor before the attempt at Cæsarean section.

I cannot resume my seat without congratulating Dr. Jaggard upon his very elaborate and learned report of his case. I think he is certainly to be thanked by this society on the production of his paper.

Professor W. W. JAGGARD, in closing the discussion, said he was grateful to the

Fellows for the kind attention given to his paper.

After answering Dr. Hoag's question, he said that he thought Dr. Parkes' notion of Cæsarean section, as expressed in his own words, was erroneous and calculated to mislead. The *technique* of the operation is apparently simple, but the dangers of shock, hæmorrhage, and sepsis are constantly present, and every minutest detail demanded the most critical attention. The terrible mortality of the operation with us in the United States abundantly demonstrates its formidable character.

Professor Charles T. Parkes read the following Report of First Fifty Operations for Ovarian Tumors.

In presenting for your consideration a tabulated list of my first fifty cases of operation done in succession for ovarian tumor, it will be my object to call attention to those only which seem to me to possess somewhat special characteristics, or have shown something unusual in their course. Not but what I believe that every case is of special interest to the operator, in so far as it furnishes him individually with useful experience and something new to cogitate over, and from which to elucidate improvements in future cases coming under his care. To mention all these circumstances would become monotonous.

In the final summing up I shall attempt to group together, in a somewhat practical way, the deductions which come to my mind as the outgrowth of this amount of work.

My work in this field commenced rather early in my professional career, and whatever success may have attended my efforts could not have been, and was not, the outcome of any special preparation. The first half-dozen operations were done before ever having witnessed the operation performed by any other operator.

In 1878 it was my privilege to see considerable of this kind of work executed by the attendants at the Samaritan Hospital, London, and other surgeons.

Since then, I have felt more at my ease in this labor, and could speak more emphatically as well as encouragingly to my patients. Before then the work had associated with it a large expenditure of force, both mental and physical, on the part of the operator, and I am free to admit that, as a rule, the patients did not get along as easily and smoothly as they have since. Still it has been my good luck not to have a very large percentage of mortality. The table shows that the second and the thirty-seventh cases died as the result of circumstances attending the operation. Two out of fifty—it is not a bad showing. If it is my good fortune to equal this percentage in the second fifty, no complaints will be heard from me.

Case I.—This lady is still living in this city. She has never borne any children. The operation was done during the third year after my graduation, and is chosen for remark, first, because the case furnishes a good illustration of the impudence and assumption sometimes displayed by young and ambitious practitioners, and which causes them at times to run where angels would fear to step even slowly. I believe I invited to be present at the operation Professors Freer, Gunn and Powell, Jackson, Bogue, and some others, and they were all on hand. It has always been a mystery to me how that operation was carried on, or finished; perhaps some who were present might be able to tell, it is impossible for me to do so. However, it is clear in my mind that there was no encouragement to me in their prognostications as to the result. They were unanimous in the assertion that the issue would be fatal. But it was not, although the patient had a hard time of it for a while. Secondly, the case is of interest in the condition which made her recovery slow and full of hazard. The fourth or fifth day showed evidences of profound septic infection. An abscess was at last discovered in the cul-de-sac of Douglas. After opening and washing it out and draining thoroughly, she passed on rapidly to a full recovery. This latter inflammation may have so changed the remaining tube or ovary as to account for the subsequent sterility. No antiseptic precautions were adopted in this case.

Case II.—This case was one of double ovarian tumor. The right cyst was free, non-adherent, and easily removed. The left was universally adherent to the left side of the abdomen, to the small intestines, spleen, and stomach. Its contents were so gelatinous that they were scooped out with the hands, and the cyst-wall was so thin that it broke down in many places during these manipulations, allowing the contents to become disseminated about and around the abdominal organs. The oozing was very free from the extensive surfaces of adhesions, and there was used a solution of persulphate of iron to check it. This remedy is a very unpleasant one to employ; this was the first and last case in which I have made use of it. The abdomen was washed and cleaned, as I thought, thoroughly.

A large-sized rubber drain was carried to the bottom of Douglas' cul-de-sac. The outer end of it was connected with a long rubber tube, carried outside of the bed and beneath it, and submerged in a solution of carbolic acid. The patient died on the third day of acute septicemia. The drain, carried to the bottom of the receptacle, gave exit to about two ounces of the contents of the cyst. It is fair to think that this case could be managed better to-day. No special antiseptic measures were adopted.

Case IX.—This case was the first tumor with purely colloid contents which I had come across. It was with the utmost difficulty that its contents could be emptied out of the rather small incision which exposed the tumor. Still, by persevering effort, it was all dug out, and as few and recent adhesions only were found, the empty sac was pressed out and rather easily removed. I have seen such cases treated by enlarging the abdominal incision in order to turn the mass out entirely. It has struck me that this method was not as good as emptying the cyst through the small incision. As all the cases turned out *en masse* have died, that fact may have influenced the formation of the opinion expressed. The cause of death may have been something else.

Case XXVI.—This case was the youngest person upon whom I have operated. Owing to the great size of the tumor, as compared with the size of the body, she presented a very odd appearance.

In order to maintain an equilibrium while in the erect position the shoulders were thrown far back—a plumb dropped from the shoulders touched the floor six inches behind the heels.

The tumor was a dry one, and before it could be extruded the abdominal incision was prolonged above the umbilicus. For such a massive tumor the pedicle was very small as well as elongated. The weight of the tumor was fourteen pounds. The case was to me particularly interesting because it was the only one of the series in which any attempt was made to carry out *in full* all the details of a Listerian operation, including the spray. Notwithstanding all this, an abscess formed in the left iliac fossa, which delayed the recovery for weeks and placed her life in great danger, especially as, even after it was opened externally, it seemed to empty internally into the bladder; large quantities of pus were passed from that viscus.

At the end of six weeks she had entirely recovered. This was the only case in which I have been haunted with the fear that I might have left some foreign body in the abdominal cavity, such as a sponge or a pair of forceps.

The sequelæ showed it to be a groundless fear fortunately. The subsequent history of this case is also interesting.

At the end of two years she again came under my care with the abdomen distended with a large growth.

This secondary growth commenced in the upper zone of the cavity and in its development increased downward. It was diagnosed to be post-peritoneal, on account of the crackling which could be produced by manipulation in circumscribed spots over the surface of the tumor. The noise was evidently produced by the displacement of intestinal gases over limited spaces.

Upon opening the abdomen, the mass was found entirely behind the peritoneum, which was opened posteriorly and the tumor easily enucleated. As far as could be determined, the mass grew from the lesser end of the pancreas.

She did not survive the shock of the operation but a few hours. The mass proved to be sarcomatous. A post-mortem examination showed no traces of even the stump of the first tumor.

Dr. Fenger was present at the first operation and pronounced the tumor to be a heterologous growth.

With an entire absence of any remnants or signs of the primary tumor, it seems rather difficult to trace any connection between it and the secondary manifestation.

Case XXXV.—This was a case of double papillomatous ovarian cyst, in which the tumors had become intimately adherent, filled up the pelvis entirely, so as to absolutely conceal the womb and bladder. The cysts had ruptured so that in themselves they were small. The abdomen was distended with an immense quantity of free fluid. They were freely enucleated from their bed and from the surface of the uterus and bladder and removed. There was left as the result of this extensive peeling a surface coequal in size with the capacity of the pelvic basin. After securing the pedicles and a few spurting arteries, the bleeding was easily staunched and showed no disposition to return after the introduction of a large drainage-tube to the bottom of the cavity and closing of the abdomen. I have never seen a larger flow of serum from a drainage-tube than followed in this case for several days. Fortunately no infection of the general peritoneal surface had occurred, so that the lady recovered very rapidly and is well and strong to-day. The free drainage in this case, I have no doubt, contributed very greatly to the easy recovery. Without it I believe the patient would have been suffocated by accumulation of serum, certainly no powers of elimination could have removed the amount of fluid drained.

Case XXXVII.—This case was certainly the worst I have ever met with so far as extent and firmness of adhesions go. It was universally adherent to the abdominal walls, the small intestines, the bladder, the uterus, the under surface of the liver, and to the stomach. In fact, at only one place was a space as large as the surface of the hand untrammeled by adhesions. This was around and about the pedicle. After separating the attachments to the abdominal walls down to this space on the right side, the pedicle was ligated and divided. It seemed impossible to reach the limits of the tumor from the anterior surface upwards, so after securing the pedicle the sac was turned upwards and the separation of the adhesions carried on from behind. It was a very slow and tedious piece of work, separating coil after coil of small intestine. When the stomach was reached, the adhesions were found so firm and extensive that it was deemed best to leave a large piece of the external layer of the sac-wall attached thereto, rather than to try to separate them. This was accordingly done. All of the tumor except the piece left on the stomach was finally removed. There was not an excessive amount of bleeding, and the abdominal cavity was readily cleansed and a drain put in.

The operation was done in Nebraska, and I had to leave the patient within two hours after the operation was finished. She was then in good condition. She died on the sixth day, as the doctor in charge wrote me, with all the symptoms of unrelieved obstruction of the bowels. Perhaps if the abdomen had been re-opened early in the manifestations of these symptoms, the obstruction might have been overcome, but this can only be a supposition. This was the second and last death in the series of fifty cases.

CASE XXXVIII.—The tumor in this case was accidentally discovered while operating upon a growth developed in the abdominal walls over the neighborhood of the gall-bladder and from which there was removed a gall-stone of considerable size. Some months after the recovery from this operation, laparotomy was done for the small tumors which filled the pelvis and were developed from both ovaries—the right one much larger than the left, but both small. They proved to be ruptured cysts, showing papillomatous degeneration. Some six weeks after this operation, after the wound had united well and recovery seemed established, she developed increasing symptoms of bowel obstruction. Examination of the rectum revealed a cancerous mass at the upper end of the rectum, probably also involving the sigmoid flexure. She was anæsthetized and the narrowed channel well dilated, sufficiently at least to relieve the accumulated contents. Still the patient gradually emaciated, and at the end of a week or ten days succumbed to the effects of the complication.

CASE XLI is only remarkable from the age of the patient—78 years old—and the perfectly uneventful recovery after the operation. Looking back at the case, I remember the impression made upon me at the time was that she was the most contented patient ever under my care.

No anxiety or worry of any kind was manifested. Everything done was good enough for her and gracefully accepted. The querulousness and disposition to be exacting sometimes supposed to go with old age was never displayed. I am quite sure her peaceful disposition had much to do with her speedy and happy recovery. I have since operated on a lady 68 years old for an ovarian cyst, and the results were nearly alike. So far as these two cases are of account, nothing whatever occurred that would make quite old age militate against the performance of the operation. The shock did not seem so great as in many other easier cases in younger patients, and the reaction was quite as prompt and harmless.

The case under consideration was a large multilocular cyst with thick walls and septa. The previous tappings had not apparently left any unfavorable conditions in their wake. The adhesions present gave no noticeable trouble.

CASE XLII.—This was my first case of twisted pedicle. The inflammatory symptoms—peritonitis—high fever, and extreme prostration had existed several days before the patient came under my care. Operation was advised and done immediately. The bad symptoms subsided at once, and the recovery was uneventful.

There followed in due course quite a large ventral hernia, although primary union occurred in the wound, at least in the skin.

It is possible that the usual care in picking up surely each layer of the abdominal wall was not followed in closing the incision, but I was not aware of leaving anything undone in that respect at the time of the operation. The diagnosis of twisted pedicle was based upon the previous existence of the tumor, its sudden and rather rapid enlargement, extreme tenderness of the tumor followed by the usual symptoms of peritonitis and constitutional manifestations of early and severe character. There was present also a free flow of dark blood from the uterus, commencing with the first symptoms and persisting.

CASE XLV was the second tumor removed having very thick colloid contents. It was perfectly symmetrical and free from adhesions of any importance.

The contents were extremely tenacious, and their removal to diminish the size of the cyst was attended with extreme difficulty. The cyst was held very carefully against the edges of the abdominal incision during these efforts, in order to avoid the entrance of any of the stuff into the peritoneal cavity. It is much easier to keep it out entirely than it is to get it out after it has once gained admission into that cavity. On two occasions the leaving of a very little of this material in the cavity inadvertently has given rise to serious complications in my experience.

CASE LX.—This case was also one of twisted pedicle; the symptoms of rapid increase in size, tenderness, and uterine hæmorrhage coming on suddenly and persisting, with developing peritonitis, were plainly present in the case and early operation advised. Consent to operate was not obtained readily, and when the incision was made the cyst and pedicle were found black.

Ulceration between the living and dead portion of the pedicle had well advanced at the site of the twist lowest down on the pedicle. Its complete separation was unattended with hæmorrhage. The case did well from the very first day.

REMARKS.

It has always been my aim to do every one of the operations here recorded with closer and closer attention to absolute cleanliness of person, assistants, patient, and of appliances.

As time passed along, more experience gained, and complicating difficulties traced to their cause, after suffering manifold mental worry over them, this aim has been better and more certainly attained, with a corresponding increase in confidence in myself and ability to make assurances to the patient with an abiding faith in their fulfillment.

There is no doubt in my mind whatever about the good done a patient by relieving her mind of doubts and nervous dread preceding the operation, as can be done by confident assertion. The success attained in ovariotomy of late years warrants an indulgence in very strong assurances on the side of recovery in all classes of cases.

The attempts to secure asepsis—to surely save one's patient from the dangers of fermentation, suppuration, and decomposition of wound secretions—brooks no neglect of any kind in the items already mentioned. It is not a pleasant thought to be forced to the conviction that you have rewarded the confidence and faith reposed in you by carrying to the afflicted one the elements which, once developed, so often destroy life, especially if the misfortune be the result of carelessness or over confidence. So nothing that is used or brought in contact with the patient should be allowed to pass without the closest inspection by the operator himself. The patient puts her life in the operator's hands, not in those of an assistant, and is entitled to the former's own care and attention to the smallest detail in the preparation of needles, forceps, and instruments of all kinds, ligatures, sponges, and dressings. It is my conviction that sponges should not be used the second time in abdominal operations, no matter how well they are cleaned. They are so difficult to free absolutely from the contamination of blood and secretions that one can scarcely be sure of them. Besides, the operation is so well paid for in most instances, and the material so cheap, that there seems no excuse to run any danger whatever.

The greatest diligence should be observed in keeping everything harmful out of the peritoneal cavity. Reference is made not so much to foreign bodies of large or small size, although such ought never to occur, as to the escape of the contents of the cyst into the cavity. To me it has always been a very difficult undertaking to clear out any such secretions, especially if they are from a cyst with sticky contents. In two cases I worked for fully an hour in my desire to be sure that all particles had been removed, and yet in both cases an abscess subsequently formed, accompanied with a formidable temperature and general exhaustion. These accumlations were fortunately found and opened. Their contents showed more or less of the same material that filled the cyst, and the trouble was evidently dependent upon its presence in the cavity. The stuff will not flow through a drain easily, so that I am not sure its use would have overcome the difficulty.

The contents can usually be kept out of the peritoneal sac by making the cyst constantly expand the edges of the abdominal incision during the necessary manipulations, by careful pressure against the tumor by an assistant.

The ligatures used have always been of carbolized silk, and they have never given rise to any trouble. In the greatest number of cases the pedicle has been clamped, the tumor removed, and the stump thoroughly cauterized down even with the clamp. Then the pedicle was sufficiently subdivided just below the clamp and ligated with silk, after which the clamp was removed and the stump dropped. I have never had, following this method, any bleeding, or been called upon to reapply the ligature, or fish up a stump out of the pelvis after it had been dropped, to stay hæmorrhage. It is the method used by Dr. Homan, of Boston.

Accidents such as indicated have hapto me when using other methods, and I have seen them occur in the hands of other operators.

Perhaps I may be pardoned for uttering a warning against using the ends of a ligature just tied for the purpose of bringing the tied tissue into view for inspection, especially against using them to in any way steady or lift the pedicle. This latter should always be fixed and manipulated with a pair of forceps fixed to its edge below the site of ligation. On more than one occasion traction on the ligature, apparently slight, has destroyed its compression and induced bleeding, or even torn it entirely loose, necessitating a tedious search for the lost stump in order to re-tie it; and one never feels as certain of the security against hæmorrhage after such an accident, aside from the delay and annoyance caused.

My experience confirms the great worth and necessity for the drainage-tube in many cases. Cases with many vascular adhesions leaving extensive oozing surfaces seem to always require the drain. Many cases would undoubtedly do better with it, even in which the raw surface is not large. One is more apt to err on the side of leaving it out than of making use of it too frequently. It takes but little over-weight of absorption and elimination of even not badly contaminated fluids to upset a patient's easy recovery, which might have all been obviated by the use of a drain for twenty-four or forty-eight hours. I have not noticed much difference in its workings, whether it be of glass or rubber; I have used both and the object aimed at was accomplished by either equally well.

The abdominal wound has always been closed with the silk suture passed carefully and carried through the different layers of the abdominal walls, including the peritoneum. It does not seem that any more satisfactory method has been advanced. It is quickly executed, and absolutely trustworthy in the vast majority of cases. Two of my cases have had ventral hernia follow; but I am inclined to think other things had something to do with the occurence of the complication, such as too early assumption of the erect position, too free motion, and discarding the abdomiual support too soon. Very few of the cases have shown any suppuration in the track of the sutures, or other complication in the line of the incision; certainly no more that six gave any trouble whatever. In very thick, fat walls, the use of three or four button-stay sutures, introduced well away from the edges of the incision, is of great advantage in maintaining the parts in close apposition and conducing to early and firm union.

In the after-treatment of the earlier cases it was the rule to use the catheter to empty the bladder six hours after operating. Quite a number of the cases developed a troublesome cystitis, and in some cases a urethritis, no matter what care was taken with the instrument or in its introduction. Of late it is not used unless absolutely required. The patient is induced to make earnest efforts at self-relief, and success generally follows these efforts, and the cystitis has ceased to be a complication.

It has become my habit not to feel concerned about a temperature up to 101° Fahr., coming during the first three or four days after an operation, if it be unaccompanied with unusual pain, headache, or anorexia. By securing a free action from the bowels by the administration of 5 grs. of hydrarg. submur., followed in due time by some saline cathartic, and urging the patient to partake freely of water, the temperature ordinarily drops to about normal in twenty-four hours. If, with a nearly normal temperature for several days after operation, it suddenly mounts to 100° or more, some complication is impending, and it must be sought for with great care. Latterly it has been a surprise to me how many of the cases go on to a safe recovery without the administration of any medicine. If sepsis is avoided, the individual's own powers of repair seem entirely competent to combat other complications with the simplest of assistance. When pain is a complication, rectal injection of the tr. opii deodorata, in full, free doses (30 drops or more), has always seemed to cause the least disturbance and accomplish the best results.

In none of these cases did there arise any necessity for re-opening the abdominal wound.

The highest temperature recorded occurred in the twenty-sixth case, in which 104° was present for several days. The abscess was found and opened and the girl got well. Cases one and nine, forty-five and forty-seven, also had abscess collections, with high temperatures. The collections of septic matter were opened where developed, and the cases finally recovered; but the complication entailed upon them a slow recovery and a weakened general condition which those escape who pass through their ordeal free from such complication.

Both with these serious conditions and other slighter ailments, less severe but absent entirely in perfectly aseptic cases, the fault must be laid upon my own shoulders. At first lack of experience, then want of attention to detail in appliances or surroundings, and finally, perhaps, over self-confidence. It is my belief that he will have the best success who is modest enough to be haunted by some doubts about himself, and so to be ever on the watch to prevent the entrance of harm from without.

The internal remedies from which the best results have been obtained for the relief of tympanitis are the spts. terebinthina and tr. nux vomica; the former to allay gaseous fermentation and as an antiseptic; the latter acting probably as a stimulant to intestinal peristalsis. It has never seemed to me that much of any good was accomplished by the rectal tube. It is not my wish to advise against its use, for many operators believe in its efficiency and use it constantly. It is quite possible that I do not use it skilfully. However that may be, I do not remember to have gained much if any good by its use. Cases in which the introduction of the rectal tube released any amount of gas could always relieve themselves by exercising a little will power, partially that of relaxation of the sphincter, mainly that of contraction of the abdominal walls. It has seemed to me that its presence in the rectum, if left there, as is practiced by some, might be of service as a foreign body in exciting peristalsis. Tympanitis, like so many other complications when they come, is usually the result of septic infection, and is best dealt with by keeping the germs away from the patient before, during, and after the operation.

The fluid I am in the habit of using for purposes of washing or irrigation is plain distilled or boiled water, with the addition of a small quantity of carbolic acid, making a solution of a strength of about two per cent. It does not seem certain that the germicidal power of this solution is of much consequence, still it does not seem worth while to dispense with it entirely. In washing out the peritoneal cavity, if occasion requires, a strong solution of boracic acid is used, and has done its work harmlessly and satisfactorily. Of course, reference is made here entirely to ovarian tumors, pure and simple. Infected cases, with pus present and other harmful fluids, require more powerful antiseptics and assiduous care in getting rid of their presence by every known means.

The fifth and thirty-fifth cases of this series showed papillomatous degeneration and rupture of the cyst wall, with the presence of extensive accumulation of ascitic fluid, rendering the diagnosis extremely uncertain. I have operated on several cases since, and they have all been difficult to diagnose and to handle. Fortunately, these two presented no secondary infection of the peritoneum, and they recovered.

The diagnosis must be made by a process of exclusion:

Heart dropsy, by its absence of facial œdema and heart lesions. Kidney dropsy, by the absence of leg œdema and the signs of kidney degeneration as shown by the microscope. Ascites, from liver or vessel obstruction, by absence of the manifestations of disease of those organs, as shown by careful physical examination and inquiry as to their usual constitutional manifestations.

Digital examination in all the cases seen by me has demonstrated the presence of a somewhat circumscribed mass in the pelvis from which the uterus could be isolated. The feel of the mass itself gives one the sensation of touching an irregular, doughy, cauliflower-like tumor. The differential diagnosis from tubercular peritonitis with ascites will be, in the absence of the tumor from the pelvis and the presence of a greater or less number of irregular, hard, perhaps movable, masses distributed through the cavity, showing involvement of the omentum.

Differential diagnosis of cancer will be probably in the fact that the latter shows less ascitic accumulation, a more easily defined mass much harder to the touch. Examined through the pelvis, every tissue is apt to show infiltration; the uterus is fixed and implicated in the growth; the roof of the pelvis everywhere hard and resisting. There will more likely be evidence of interference with the circulation of one limb, as shown by œdema confined to one side. Enlarged and tortuous external abdominal veins and more rapid and profound constitutional manifestations will be present. Even after rupture of the sac and moderate infection of the peritoneum, these cases seem to do perfectly well after operation and removal.

It seems to be of paramount importance to institute such care of the patient as will most surely prevent, diminish, or overcome the occurence of shock. After every severe operation, much can be done by the use of external warmth, and also care during the progress, by keeping wet clothes away from the body. I am still convinced of the efficacy of morphia and quinia administered half an hour or so previous to the commencement of an operation.

It can scarcely be denied that the patients do best if little, or better still, nothing, is put into the stomach for twenty-four hours or more. If introduced, the effect is merely to increase the disposition to vomit.

Judging from the results of considerably over one hundred laparotomies for different diseases, I think it proper to say that as a rule an operation for the relief of a simple ovarian tumor is about the simplest proceeding the surgeon is called upon to do in the abdominal cavity, and one from which the patient is most likely to recover.

Professor C. T. PARKES, in closing the discussion, said: There is very little to say, except, perhaps, in reference to what was

| No. of Operation. | Date. | Name. | Age. | Kind of Tumor. | Which Ovary. | Catamenia Since. | Children Before. | Children Since. | Married or Single. | Health Since. | Incision. | Rupture. | Ligatures Heard From. | Twisted Pedicle. | Weight. | Drainage. | Adhesions. | Pedicle, Kind and How Treated. | Result. | Remarks. |
|---|---|---|---|---|---|---|---|---|---|---|---|---|---|---|---|---|---|---|---|---|
| 1 | Sept., '71 | Hughes... .... | 23 | Multiloc. ......... | R | No | No | No | M | Good | S | No | Yes | No | 27 lbs. | No | Yes | Transfixed; ligated ...... | R | Ligature discharged through abscess in cul-de-sac of Douglas two weeks subsequent. |
| 2 | " '71 | *Forberger!*..... | 35 | Mul. colloid ..... | R & L | ? | Yes | ...... | M | Died | L | No | ...... | No | 43 " | / Yes | Yes | Long pedicle; transfixed and ligated. | D | Left very large; right small and single. Tapped once previously. |
| 3 | " '73 | Winchell ..... | 49 | Mul. cyst ........ | L | No | No | ? | M | Good Died 1880 | S | No | No | No | 34 " | No | Yes | Moderate pedicle; transfixed and ligated; silk. | R | Right ovary also removed; cystic degeneration. Tapped twice. |
| 4 | " '73 | Peters ......... | 37 | Broad lig. cyst... | R | Yes | No | ? | M | In 1881 Good | S | No | No | No | 22 " | No | Yes | (Enucleated) ligated; silk | R | Cæcum and colon carried upon wall of tumor some distance. |
| 5 | " '73 | O'Mally........ | 45 | Double papilloma | R & L | No | Yes | No | M | Good Died 1884 | L | Yes | No | No | 42 " | Yes | Yes | Short; transfixed; clamped, cauterized, and ligated. | R | Cyst small. |
| 6 | " '73 | Carney..... ... | 44 | Unilocular ....... | R | No | Yes | No | M | Good Died 1878 | S | No | No | No | 26 " | No | No | Fair; transfixed and ligated. | R | Contents milk-white. |
| 7 | April, '73 | McGovern . ... | 33 | Multiloc.......... | L | Yes | No | Yes | M | Good | S | No | No | No | 21 " | No | Yes | Long; transfixed and ligated. | R | |
| 8 | " '74 | Hunt .......... | 39 | Unilocular.... .. | L | ? | Yes | ? | M | Good | S | No | No | No | 33 " | No | No | Long and slender; transfixed and ligated. | R | Thick walls; chocolate-colored fluid. |
| 9 | " '74 | *Edwards!*..... | 41 | Colloid tumor ... | R | Yes | No | No | M | Good | L | No | No | No | 32 " | No | / Yes | Broad; short four sections; clamped; cautery; ligated. | R | ? |
| 10 | " '75 | Foster ..... | 52 | Multiloc ......... | R | No | 7 | No | M | Heard 3 years after, Fair | S | No | No | No | 24 " | No | Yes | Short and broad; transfixed, ligated, and cauterized. | R | |
| 11 | May, '75 | Martin......... | 57 | Broad lig......... | L | Yes | No | ? | M | Don't know | S | No | No | No | 28 " | No | No | Transfixed and ligated. | R | Enucleated. |
| 12 | Sept., '75 | McNulty ....... | 28 | Multiloc.......... | L | Yes | No | ? | M | Good | S | No | No | No | ...... | No | Yes | Ligated in many sections and divided. | R | Also removed mass of omentum |
| 13 | Dec., '75 | Jones.......... | 48 | Multiloc. cyst ... | L | ? | 5 | ? | M | 5 years after, Fair | S | No | No | No | 22 " | No | Yes | Thick; transfixed and ligated. | R | No answer to letter. |
| 14 | " '75 | Hunter ....... | 55 | Multiloc. cyst.... | R | No | 2 | No | M | Fair Died 1876 | S | No | No | No | 25 " | No | Slight | Short and thick; transfixed, ligated, and cauterized. | R | |
| 15 | Jan., '79 | Hedrick ....... | 62 | Multiloc. cyst.... | L | No | 3 | No | M | Fair 3 years after | S | No | No | No | 18 " | No | No | Moderate; transfixed, ligated, and cauterized. | R | |
| 16 | " '79 | Phillips ....... | 36 | Multiloc. cyst.... | L | Yes | No | No | M | Poor | L | No | No | No | 39 " | Yes | Yes | Broad, short and thick; transfixed, ligated, and cauterized. | R | Semi-solid contents. |

| No. of Operation. | Date. | Name. | Age. | Kind of Tumor. | Which Ovary. | Catamenia Since. | Children Before. | Children Since. | Married or Single. | Health Since. | Incision. | Rupture. | Ligatures Heard From. | Twisted Pedicle. | Weight. | Drainage. | Adhesion. | Pedicle, Kind and How Treated. | Result. | Remarks. |
|---|---|---|---|---|---|---|---|---|---|---|---|---|---|---|---|---|---|---|---|---|
| 17 | Mar., '79 | Henderson..... | 29 | Uniloc. cyst ..... | L | ? | No | ? | M | Good | S | No | No | No | 18 lbs. | No | No | Moderate; transfixed and ligated. | R | |
| 18 | Mar., '79 | Pollock........ | 31 | Multi. cyst, colloid | R | ? | ? | ? | M | Fair | L | No | No | No | 23 " | No | Yes | Medium; transfixed, ligated, and cauterized. | R | |
| 19 | June, '79 | Probiski....... | 25 | Mulit. cyst. Uniloc. cyst. | R | Yes | ? | ? | M | Fair | S | No | No | No | 38 " | No | Yes | Fair; long and broad; transfixed, ligated, and cauterized. | R | |
| 20 | " '79 | Healy.......... | 18 | Parovarian cyst.. | L | Yes | ? | 1 | S | Good | S | No | No | No | 23 " | No | No | Short and broad; ligated in four sections. | R | |
| 21 | " '79 | Dayton.... .... | 36 | Dermoid cyst .... | R | Yes | 2 | No | M | Fair | S | No | No | No | 18 " | No | Yes | Broad; transfixed; ligated in three sections. | R | Adherent portions of cyst wall, to cæcum and uterus left |
| 22 | Nov., '79 | Johnson.... ... | 43 | Parovarian cyst.. | L | ? | No | No | M | Good 5 years ago. | S | No | No | No | 23 " | No | No | Long and slim; transfixed and ligated. | R | |
| 23 | " '79 | Jones.......... | 23 | Multiloc. cyst.... | R | Yes | No | No | S | Good | S | No | No | No | 19 " | No | Yes | Broad; ligated in three sections and cauterized. | R | |
| 24 | " '79 | Mason.... ..... | 65 | Multiloc. cyst.... | L | No | 3 | No | M | Died 2 years after. F'ir to death. | S | No | No | No | 19 " | No | Yes | Broad; transfixed and ligated. | R | |
| 25 | '80 | Mastin......... | 39 | Parovarian cyst.. | R | Yes | ? | ? | M | 1883 Good | S | No | No | No | 23 " | No | No | Broad; ligated in four or five places. | R | Enucleated. |
| 26 | Aug. '81 | M. Herman.... | 7 | Sarcoma...... .... | R | No | No | No | S | Good | L | No | Yes | No | 14 " | No | Yes | Long; transfixed and ligated. | R | Died three years subsequent; return of disease in pancreas |
| 27 | June, '82 | Peterson....... | 24 | Multiloc. cyst .... | R | No | No | ...... | S | Good | S | No | No | No | 35 " | No | Yes | Long and slender; transfixed, ligated, and cauterized. | R | |
| 28 | Feb., '83 | Allen, Mary.... | 18 | Multiloc. cyst.... | R | Yes | No | No | S | Good | S | No | No | No | 28 " | No | No | Broad and short; transfixed, ligated in four sections, and cauterized. | R | |
| 29 | June, '83 | Connelly, Bdgt. | 47 | Multiloc. cyst.... | R | No | Yes | No | M | G'd to death. | S | No | No | No | 34 " | No | Yes | Four inches long and three broad; transfixed, ligated, and cauterized. | R | Died 1885. |
| 30 | " '83 | Mackey, Maggy | 23 | Simple cyst ...... | R | Yes | No | No | S | Good | S | No | No | No | 16 " | No | No | Moderate; transfixed, ligated, and cauterized. | R | |
| 31 | " '83 | O'Neil......... | 48 | Multiloc......... | R | No | No | No | M | Good | S | No | No | No | 37 " | No | Yes | Broad and short; transfixed, clamped, cauterized, transfixed, and ligated. | R | |
| 32 | '84 | Dwyer, Marg... | 46 | Dermoid cyst.... | L | No | Yes | No | M | Fair | L | No | No | No | 18 " | Yes | Yes | Short; transfixed, ligated, and cauterized. | R | Bone, teeth, hair, etc. Died in 1886. |
| 33 | '84 | Haggerty Nellie | 19 | Simple cyst....... | R | ? | No | ? | S | Good | S | No | No | No | 82 " | No | No | Broad and thin; transfixed, ligated, and cauterized. | R | |
| 34 | Sept., '84 | Riley, Mary.... | 25 | Multiloc. cyst.... | L | Yes | No | No | S | Fair | L | No | No | No | 27 " | No | Yes | Thick and broad; transfixed and ligated in three sections. | R | |

| No. of Operation. | Date. | Name. | Age. | Kind of Tumor. | Which Ovary. | Catamenia Since. | Children Before. | Children Since. | Married or Single. | Health Since. | Incision. | Rupture. | Ligatures Heard From. | Twisted Pedicle. | Weight, | Drainage. | Adhesion. | Pedicle, Kind and How Treated. | Result. | Remarks. |
|---|---|---|---|---|---|---|---|---|---|---|---|---|---|---|---|---|---|---|---|---|
| 35 | '84 | *Emerson!* | 33 | Double papillomatous cyst | R & L | Yes | No | No | M | Good | S | Yes | No | No | 38 lbs. | Yes | ! *Yes* | No pedicle; everything ligated and cauterized. | R | Large amount of fluid drained off. |
| 36 | Nov., '85 | Murphy | 44 | Multiloc. | R | No | 5 | No | M | In 1886 Good | S | No | No | No | 52 " | No | Yes | Narrow and short; transfixed, ligated, and cauterized. | R | Catamenia ceased two years previous. |
| 37 | June, '85 | *Rush!* | 43 | Multiloc. cyst | R | No | Yes | No | M | Died | S | No | No | No | 40 " | No | *Yes* | Transfixed and ligated. | D | Death in six days from obstruction of bowels. |
| 38 | April, '85 | *Piper!* | 51 | Double ovarian | R & L | No | Yes 5 | No | M | Poor. Died 2 mos. | S | No | No | No | 4 " | Yes | Yes | Short; transfixed, ligated, and cauterized. | R | Died two months after; cancer of rectum. |
| 39 | Sept., '85 | Morrill | 29 | Dermoid cyst | L | Yes | No | No | M | Good | L | No | No | No | 18 " | No | Yes | Broad and thin; transfixed and ligated. | R | Surface of uterus sewed over for hæmorrhage. |
| 40 | " '85 | Patterson | 37 | Multiloc. cyst | L | Yes | Yes 2 | No | M | Fair | S | No | No | No | 35 " | No | No | Long; transfixed, ligated, and cauterized. | R | |
| 41 | '86 | Holland | 78 | Multiloc. cyst | R | No | Yes 8 | No | M | For 1 year Fair. | L | No | No | No | 43 " | Yes | Yes | Broad; ligated in four sections and cauterized. | R | Tapped three times. Died one year subsequent. |
| 42 | Jan., '86 | *Ripkow!* | 49 | Multiloc. cyst | R | Yes | Yes | No | M | Good | S | No | No | Yes | 25 " | No | Yes | Long and narrow, and twisted three times; suppuration commenced; ligated below line of demarkation. | R | Uterine hæmorrhage previous. Ventral hernia subsequent. |
| 43 | Aug., '86 | Lewis | 31 | Multiloc. cyst | L | No | No | No | M | Poor | L | No | No | No | 22 " | No | Yes | Short; transfixed, ligated, and cauterized. | R | |
| 44 | '86 | Meehan | 25 | Uniloc. cyst | L | Yes | No | No | S | Good | S | No | No | No | 26 " | No | Yes | Transfixed, ligated, and cauterized. | R | Peeled out. |
| 45 | '86 | *Bick, Honora!* | 23 | Colloid | R | ? | Yes 2 | No | M | Good | L | No | No | No | 27 " | No | Yes | Fair; transfixed, ligated, and cauterized. | R | Dug out before removal. Twenty-two stitches. |
| 46 | June, '86 | Anderson | 43 | Uniloc. cyst | L | No | No | No | M | Fair | S | No | No | No | 30 " | No | Yes | Slender; transfixed, ligated, and cauterized. | R | Both ovaries removed. R. ovary diseased. |
| 47 | '86 | Conroy | 52 | Multiloc. colloid. | R | Yes | Yes 12 | No | M | Good | S | No | No | No | 26 " | No | No | Broad and short; ligated in three sections. | R | |
| 48 | '87 | Mitchell, Mag. | 13 | Multiloc. cyst | R | Yes | No | ? | S | Good | S | No | No | No | 18 " | No | No | Broad and long; transfixed, ligated, and cauterized. | R | |
| 49 | '87 | Muldahl | 37 | Multiloc. | R | Yes | No | No | M | Good | S | No | No | No | 36 " | No | Yes | Long and thick; transfixed, ligated, and cauterized. | R | |
| 50 | '87 | *Hamilton!* | 44 | Multiloc. | R | No | Yes 1 | No | M | Good | S | No | No | Yes | 21 " | No | Yes | Twisted and long; transfixed, ligated, and cauterized. | R | Uterine hæmorrhage and peritonitis previous. |

said about the simplicity of the operation. I tried to make it plain that in reference to many other operations that are done in the abdominal cavity it is simple, not in reference to all operations. Surgeons in all parts of the country are doing ovariotomy, and the result is favorable in most of these cases; it must be a simple operation or the results would not be so favorable.

I believe I was right in Dr. Jaggard's case in saying that the technique of Cæsarean section is simple. It is not simple for the patient and never will be. The only reason why it can ever be safe rests in the employment of aseptic surgery. So I believe that laparotomy for simple ovarian tumor, compared with other operations the surgeon is called upon to do in the abdominal cavity, is the simplest.

